# The Significance of Thyroid Hormone Receptors in Breast Cancer: A Hypothesis-Generating Narrative Review

**DOI:** 10.3390/curroncol31050176

**Published:** 2024-04-23

**Authors:** Trinity Quan, Jessica Cockburn, Sukhbinder Dhesy-Thind, Anita Bane, Hon Leong, Christopher Geleff, Catherine Devion, Noor Ajel, Katarzyna J. Jerzak

**Affiliations:** 1 Division of Medical Oncology, Sunnybrook Odette Cancer Centre, University of Toronto, Toronto, ON M4N 3M5, Canada; tquan5@uwo.ca (T.Q.); 20na30@queensu.ca (N.A.); 2 University Health Network, Toronto, ON M5G 2C4, Canada; jess.cockburn@uhn.ca (J.C.); anita.bane@uhn.ca (A.B.); 3 Department of Oncology, Juravinski Cancer Centre, 699 Concession St, Hamilton, ON L8V 5C2, Canada; dhesy@hhsc.ca; 4 Department of Medical Biophysics Temerty, Faculty of Medicine, University of Toronto, Toronto, ON M4N 3M5, Canada; hon.leong@sri.utoronto.ca (H.L.); c.geleff@queensu.ca (C.G.); 5 Sunnybrook Research Institute, Sunnybrook Health Sciences Centre, Toronto, ON M4N 3M5, Canada; 6 Library Services, Sunnybrook Health Sciences Centre, Toronto, ON M4N 3M5, Canada; catherine.devion@sunnybrook.ca

**Keywords:** thyroid receptor beta, breast cancer, biomarker

## Abstract

Background: Breast cancer (BC) is frequently diagnosed among Canadian women. While targeted therapies are available for most BC patients; treatment resistance is common and novel therapeutic targets are of interest. Thyroid hormones (TH) bound to thyroid hormone receptors (THR) influence cell proliferation and differentiation; they are also involved in the growth and development of normal breast tissue. Evidence suggests that THRβ is a tumor suppressor in various solid tumors. Purpose: This narrative review discusses retrospective studies regarding the clinical relevance of THRβ as a potential prognostic biomarker and therapeutic target in BC. Methods: We consulted with an information specialist to develop a search strategy to find all literature related to THRα expression as a potential prognostic and therapeutic biomarker in breast cancer. The primary search was developed for Medline and translated to Embase. The searches were conducted on the Ovid platform on 18 August 2023. Results: Across seven retrospective studies identified, several have shown an association between higher THRβ1 expression with a lower risk of BC recurrence and with longer overall survival. Conclusions: Some evidence suggests that THRβ expression is associated with a lower risk of BC recurrence and death. Validation of THRβ as an independent prognostic biomarker and possible predictive biomarker of response to endocrine therapy and/or chemotherapy is of interest. Given that THRβ is upstream of the AKT/PI3K pathway, its potential as a predictive biomarker of response to AKT inhibitors and/or PI3K inhibitors may also be of value. Finally, the potential re-purposing of THRβ agonists as anti-cancer agents warrants investigation.

## 1. Introduction

Breast cancer (BC) is the most frequently diagnosed cancer among women, with an overall lifetime risk of approximately 1 in 8 in North America [1]. Four major molecular subtypes of BC have been reported, including luminal A, luminal B, human epidermal growth factor receptor 2 (HER2) positive and triple-negative subtypes [2]. While targeted therapies are available for most patients with BC, particularly those with estrogen receptor (ER) and/or progesterone receptor (PR) positive disease as well as those with HER2+ BC, resistance to treatment is common and novel therapeutic targets are of interest [3]. In addition, biomarkers above and beyond those defining molecular subtypes of BC are required to better assign prognosis and predict therapeutic response. 

**Figure 1:**
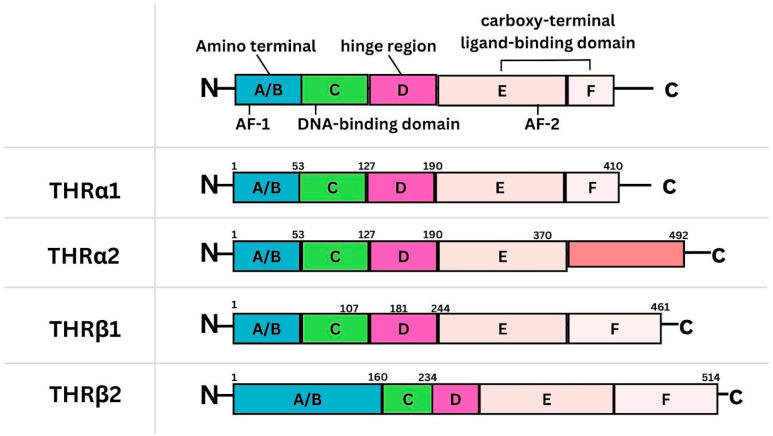
Chromosome 17 contains the THRA gene encoding THRα1 and THRα2 [4]. THRα2 holds an extra carboxyl-terminal which inhibits T3-mediated signaling and tumor growth [5]. Chromosome 3 contains the THRB gene encoding THRβ1 and THRβ2 [4].

The role of thyroid hormone receptors (THR) in the development and biology of BC has been examined in recent years. There are two known genes that encode THRs, THRA on chromosome 17 and THRB on chromosome 3, which correspond to the THRα and THRβ proteins, respectively (Figure 1) [4]. THRα is a nuclear receptor protein that has two isoforms known as THRα1 and THRα2. The THRα1 isoform is able to bind triiodothyronine (T3) and thyroid response elements (TREs), whereas, the THRα2 isoform does not bind to T3 and weakly binds to TREs [1]. While the clinical significance of THRα has not been well defined, literature suggests that THRα1 is tumor-promoting while THRα2 has opposite effects on tumor growth and proliferation. This difference in function is attributed to the fact that THRα2 holds an extra carboxyl-terminal portion, which inhibits T3-mediated signaling and tumor growth [2]. Furthermore, the inhibitory effect of THRα2 may also be attributed to its inability to bind to T3 thereby, antagonizing the action of THRα1 [5].

Comparatively, THRβ isoforms (β1 and β2 isoforms) are splice variants of the same gene [6]. The THRβ1 isoform is more widely expressed and is involved in regulating the expression of certain genes that are sensitive to thyroid hormones [6]. THRβ1 is found in the brain, heart, liver and other organs, primarily functioning in areas of metabolism, growth and development [7]. Conversely, expression of the THRβ2 isoform is contained in the pituitary, triiodothyronine-responsive neurons, the developing inner ear, and the retina [6]. Consequently, THRβ2 is primarily responsible for mediating the effects of brain development and function, playing a role in neuronal differentiation, myelination, and synaptic plasticity [7]. THRβ has been shown to be a well-characterized tumour suppressor in pre-clinical models of various solid tumors [8]. The tumor suppressive action of THRβ has also been supported in clinical literature, with several reports demonstrating favorable outcomes associated with THRβ expression in BC [9,10,11,12]. 

**Figure 2:**
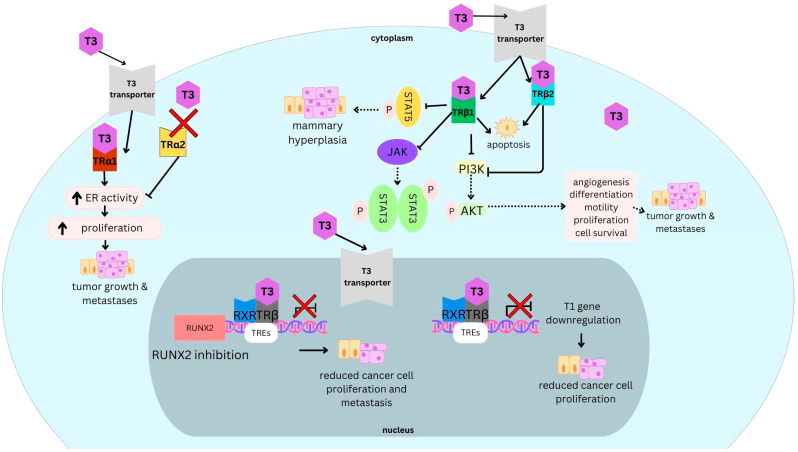
Cytoplasmic signalling pathways of thyroid hormones.

Thyroid hormones may induce their effects through genomic and non-genomic actions. Their genomic actions involve the primary interaction of T3 with nuclear THR and the subsequent interaction with specific DNA sequences known as thyroid hormone response elements (TREs) to regulate the transcription of target genes (Figure 2). In this context, THRβ acts as a transcription factor, modulating gene expression in response to thyroid hormone levels. There is also evidence to suggest that THRβ can localize to the cytoplasm where THRβ may be involved in non-genomic signaling pathways. These pathways often involve rapid responses to thyroid hormone stimulation, which may not involve changes in gene expression. Instead, cytoplasmic THRβ may interact with other signalling molecules or pathways to mediate effects such as cellular proliferation, differentiation, or metabolism [13].

The study of THRs in BC adds a layer of complexity to an already heterogeneous disease as it is necessary to consider the different receptors and their splice variants as well as the modulated effect of TH itself. For instance, the PI3K pathway is one of the signalling pathways inhibited upon T3 interacting with THRβ. Downstream signalling of the PI3K pathway has oncogenic effects, particularly through the phosphorylation of AKT, which in turn phosphorylates targets like mTORC2, a central regulator of cell metabolism, growth, proliferation, angiogenesis, and survival [8,14]. THRβ regulates the activity of the JAK-STAT pathway, ultimately leading to an increase in apoptosis and a reduction in tumor size and proliferation [15]. However, it is important to note that very few studies differentiate between THRβ isoforms and their respective roles in signalling cascades.

Multiple studies have also observed an enhanced sensitivity to chemotherapeutics upon ligand-THRβ activation [8]. In addition, an association between THRβ with longer disease-free and overall survival has been reported [9,10,11,12]. In an attempt to clearly describe the complex nature of THRs and their roles in BC, this narrative review will summarize and describe the findings of studies that investigate the clinical relevance of thyroid hormone receptors, with a focus on THRβ. 

## 2. Materials and Methods

We consulted with an information specialist to develop a search strategy to find all literature related to THRα and THRβ expression as potential prognostic and therapeutic biomarkers in breast cancer. The information specialist developed a strategy using keywords and controlled terms using studies provided by the authors that were known to meet inclusion criteria. The primary search was developed for Medline and translated to Embase. The searches were conducted on the Ovid platform on 18 August 2023. The reproducible search strategies are available in Table S1.

## 3. Results

Seven studies were identified in total, including seven retrospective studies with patient cohort sizes ranging from n = 82 and n = 1752, reflecting 3221 individual patients. 

### 3.1. Patient Cohort and Clinical Data

The characteristics of patient cohorts in the included studies are detailed in Table 1. The majority of the studies consist of patients with hormone receptor-positive, sporadic breast cancers, although Heublein et al. included patients with BRCA1 gene mutations [9,10,11,12]. The expression of THR was assessed retrospectively to interrogate associations with patient outcome. It is notable that three of the seven studies did not account for the breast cancer therapeutics administered during the period of the study as potential confounding variables in the statistical measures of association between THR expression and patient outcome [9,10,11]. In the second largest reported cohort, an association between THRβ expression and longer survival was observed, even after adjustment for age, tumor size, nodal status, chemotherapy, hormone therapy, radiotherapy, surgery, and ER status in a multi-variable model with a HR of 0.32 (95% CI 0.11–0.94), *p*  =  0.04. This suggests that an independent association exists between THRβ expression and longer overall survival [4,11].

### 3.2. Expression of THRβs and Their Predominant Localization in Human BC Cells 

Using immunohistochemistry, Ditsch et al. confirmed the positive nuclear expression of THRβ and their respective isoforms in a cohort of BC patients using specific monoclonal or polyclonal antibodies (Table 2) [10]. 

**Figure 3:**
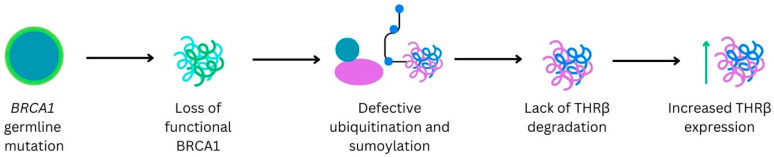
BRCA1 germline mutations association with increased THRβ.

Heublein et al. also performed immunohistochemistry to evaluate the expression of THRβ using anti-THRβ polyclonal antibodies (Table 2) in a patient cohort with BRCA1-associated (n = 38, 31%) and sporadic BC (n = 86, 69%). THRs were found to be expressed in the BC tissues in a nuclear fashion. Interestingly, 22% of sporadic breast cancers stained positive for THRβ, while a much higher proportion (53%) of the BRCA1-mutated tumors stained positive for THRβ. The rationale for these findings involves the understanding of the wildtype BRCA1 protein and its function in protein degradation via ubiquitination and sumoylation of nuclear receptors [18]. It has been hypothesized that mutations in BRCA1 (and thereby loss of functional BRCA1) decrease the degradation of THRβ and thereby contribute to THRβ over-expression [12] (Figure 3).

Shao et al. found that nuclear staining of THRβ1 was significantly stronger in intensity than cytoplasmic staining. 60% of their patient cohort had positive expression of nuclear THRβ1, and 43% had positive expression of cytoplasmic THRβ1. The ratio of nuclear versus cytoplasmic staining was determined using polyclonal antibodies (Table 2) in immunohistochemical analysis of all 263 tumors stained. 44% of tumors had equal nuclear and cytoplasmic expression, 30% had greater nuclear expression and 26% had greater cytoplasmic staining [9]. 

Ditsch et al., Shao et al., and Heublein et al. assessed the expression of THRβ1 using immunoreactive scores (IRS). The IRS score was calculated by multiplying the percentage of positively stained cells by the optical staining intensity. The percentage of positively stained cells was scored as 0 (no staining), 1 (≤10% of stained cells), 2 (11–50% of stained cells), 3 (51–80% of stained cells), and 4 (≥81% of stained cells). The optical staining intensity was graded as 0 (negative), 1 (weak), 2 (moderate), and 3 (strong) [9,10,12]. In the study conducted by Ditsch et al. and Heublein et al., IRS scores of 0–1 were classified as THR negative, and scores of 2–12 were classified as THR positive for the survival analyses [10,12]. However, in the study by Shao et al. an IRS score of 0 was classified as THR negative, and any scores greater than 0 were classified as THR positive [9]. All three of these studies found that THRs were predominantly expressed in the nuclei of malignant breast tumors [9,10,12]. 

Another study performed by Jerzak et al. established that the THRβ1 isoform specifically is predominantly expressed in the cytoplasm of breast cancer cells (Table 2). An immunohistochemical analysis using the SC-737 antibody from Santa Cruz Biotechnology was done to determine the THRβ1 expression. Allred’s method was used to score the receptor expression levels. Scores ranging from 0 to 8 were obtained by adding the intensities of staining to the percentage of cells that were stained. The intensity of staining was scored as 0 (absent), 1 (weak), 2 (moderate), and 3 (strong) and the percentage of cells stained was scored as 0 (none), 2 (1–10%), 3 (11–33%), 4 (34–66%), and 5 (67–100%). The score of 4 was used as a cut-point to differentiate between low (<4) and high (>4) THRβ1 expression. It was found that 40% of 796 BC patients had high THRβ1 expression, and 60% had low expression; in all cases, the predominant THRβ1 localization was in the cytoplasm [11]. 

### 3.3. Patient Prognosis and Clinicopathological Parameters According to THRβ1 Expression

Given the biology of THRs and the fact that they are accessible targets, it has been suggested that THRs may play a role as prognostic and/or predictive biomarkers as well as therapeutic targets [10,12]. Various studies have indicated that THRβ1 is prognostic in that its expression is associated with longer disease-free survival in patients with early BC [12,19] (Table 3). Ditsch et al. found a significant association between positive THRβ1 expression with smaller tumor size (*p* = 0.009) and a positive association between THRβ1 expression and positive ER/PR status (*p* = 0.025). A trend for an association between THRβ1 expression and longer disease-free survival (*p* = 0.082) was found but not for overall survival (*p* = 0.174). There were no significant differences in disease-free survival or overall survival between patients with positive or negative IRS scores for THRβ2 (disease-free survival: *p* = 0.830 and overall survival: *p* = 0.174) [10].

Contrary to the findings from Ditsch et al., Shao et al. performed multivariate analyses for nuclear and cytoplasmic localization of THRβ1 and various clinicopathological parameters, including age at the time of diagnosis, tumor size and breast cancer subtype. It was found that nuclear localization was associated with a shorter overall survival (*p* = 0.0004) and cytoplasmic expression was associated with a longer overall survival (*p* = 0.048). Shao et al. also found that cytoplasmic THRβ1 was associated with longer survival (*p* = 0.015) whereas nuclear THRβ1 was associated with shorter survival (*p* = 0.038); this association between cytoplasmic THRβ1 expression and favorable prognosis was shown in ER+ tumors (*p* = 0.021) but not in ER- tumors (*p* = 0.161). Interestingly, both nuclear and cytoplasmic THRβ1 expression were associated with expression of CD133 (a marker of cancer stem cells) and N-cadherin (a marker for epithelial-to-mesenchymal transition). However, only the cytoplasmic form of THRβ1 was associated with positive HER2 expression and a cellular marker for proliferation, Ki67 [9].

The analysis performed by Jerzak et al. identified an association between high THRβ1 expression and favorable tumor characteristics such as ER+ status, small tumor size, and node-negative status in early BC. A significant association between high cytoplasmic THRβ1 expression with longer BC-specific survival (*p* < 0.0001) was found and maintained in a multivariable model adjusting for age, tumor size, nodal status, ER status, and treatment variables. It is important to recognize that the association found between THRβ1 expression and BC-specific survival was found only among patients with ER+ tumors and not among those with ER- disease; further, ER+ tumors were more likely to have high THRβ1 expression than ER- tumors (92.4% vs. 7.6%, *p*  <  0.0001) [11]. In another study performed by Jerzak et al., high THRβ1 expression was also associated with longer overall survival [4].

The prognostic significance of THRβ in patients carrying a BRCA1 mutation and in patients with sporadic BC was determined by Heublein et al. Results demonstrated that THRβ was more frequently expressed in BRCA1-associated BC compared to sporadic BC. THRβ positivity in BRCA1-associated BC cases was found to be a positive prognostic biomarker for five-year (*p* = 0.007) and overall survival (*p* = 0.026). Heublein et al. found that the activation of THRβ resulted in a down-modulation of the gene encoding tumor-promoting β-catenin, CTNNB1. It was also determined that the expression of THRs in 9 out of 12 triple negative BCs (TNBCs) was highly sensitive to THR modulation in BRCA1 mutant HCC3153 cells. However, in this study, there was no significant association found between THRβ expression and patient prognosis in patients with sporadic BC [12].

Multivariate analyses performed by Shao et al., Ditsch et al., and Jerzak et al. demonstrated that higher THRβ1 expression (irrespective of intracellular location) is associated with significantly longer overall survival in patients diagnosed with sporadic BC with a hazard ratio of 0.55, 0.83, and 0.61, respectively [9,10,11]. Shao et al. and Jerzak et al. found that favorable prognostic associations were associated with cytoplasmic THRβ1 expression [9,11] whereas Ditsch et al. found that nuclear THRβ1 expression was associated with favorable patient outcome [10]. 

Muscat et al. evaluated 66 individual cases of primary invasive ductal carcinoma to determine the prognostic value of several nuclear receptors including THRβ in BC. An inverse association between THRβ expression evaluated via a gene expression analysis and histological grade was identified (*p* = 0.001); further, THRβ was associated with longer metastasis-free survival in tamoxifen-treated patients in a Cox regression hazards model (*p* = 0.001) [16]. 

Finally, Gu et al. observed an association between longer DFS in 66 patients with TNBC and high THRβ mRNA levels [17]. Furthermore, the half-maximal inhibitory concentration (IC50) values in THRβ knockdown cells after treatment with docetaxel and doxorubicin were analyzed to determine the role of THRβ as a potential predictive biomarker of response to chemotherapy in TNBC. Low levels of THRβ were associated with enhanced resistance to both chemotherapeutic drugs in HCC2185 and HCC202 cell lines. Mechanisms underlying the THRβ knockdown-induced resistance were thought to be due to reduced ability of chemotherapy to induce cellular apoptosis when THRβ levels were low [17]. 

### 3.4. Thyroid Hormones within Patient Cohorts

The activation of THRβ is dependent on the receptor’s binding affinity and on the availability of its associated ligand, THs. Moeller et al. found that high levels of thyroid hormones were associated with advanced clinical stages of BC [19]. It is known that a negative relationship exists between nuclear saturable high affinity binding sites of THs and the lymph node status of BC patients [8]. The studies mentioned in this review briefly discuss the importance of THs. However, most of the studies did not investigate ligand availability among their respective patient cohorts. Heublein et al. did, however, assess the thyroid stimulating hormone (TSH), fT3, and fT4 serum levels in their participating BC patients and quantified these levels at the time of the initial diagnosis. No relation between THR expression and circulating hormone levels were found in this analysis [12].

## 4. Discussion

While targeted therapies are available for most patients with BC, particularly those with ER and/or PR positive disease and those with HER2+ BC, resistance to treatment is common and novel therapeutic targets and biomarkers are of interest. As outlined in this review, THRs play a role in the regulation of many genes, including some that are involved in cell differentiation, proliferation, and apoptosis [16,20]. Further, as a tumor suppressor, high expression of THRβ1 has been associated with improved BC-specific survival and its downregulation of the JAK/STAT/Cyclin D pathway is a well-recognized mechanism of endocrine resistance [15]. However, the ability of THRβ1 to predict response to endocrine therapy among patients with hormone receptor-positive BC has not been evaluated. To address this question, expression of THRβ1 expression among patients who did versus did not receive endocrine therapy would need to be determined, ideally in the setting of a randomized trial. This warrants evaluation because a high proportion of patients with hormone receptor-positive breast cancer will experience a distant relapse, even after completing a 5-year course of adjuvant endocrine therapy [21]. A more accurate signature to identify patients at particularly high risk of relapse would be of value and may help to identify patients who would benefit most from additional adjuvant therapies, such as CDK4/6 inhibitors.

Given that THRβ is upstream of the PI3K/AKT pathway, it may also be evaluated as a possible biomarker of response to PI3K inhibitors and/or AKT inhibitors among patients with metastatic breast cancer. For example, in the CAPitello-291 study, the AKT inhibitor capivasterib demonstrated a 6.4-month progression-free survival (PFS) among patients with an alteration in the PI3K/AKT pathway but only a 1.6-month PFS among those confirmed not to have an alteration [22]. In addition, in the SOLAR-1 trial of alpelisib in addition to fulvestrant versus fulvestrant alone for patients with PI3K mutated, HR+, HER2-ve metastatic breast cancer, the absolute PFS benefit associated with the use of alpelisib was 5.3 months (11 months vs. 5.7 months, *p* < 0.001) [23]. Whether additional biomarkers such as THRβ could further tailor the clinical use of these agents requires further investigation. Further, whether thyroid receptors could contribute to a robust biomarker signature of endocrine sensitivity after progression on upfront endocrine therapy plus CDK4/6 inhibition would be of interest. This is due to large heterogeneity in response to 2nd and subsequent line endocrine approaches for patients with HR+ disease [23].

In addition to HR+ disease, increasing THRβ levels were associated with improved outcomes among 1752 patients with TNBC [17]. Further, Gu et al. demonstrate that low THRβ expression results in reduced apoptosis (and thereby resistance to) chemotherapy in TNBC cell lines [17]. Hence, there is a rationale to evaluate THRβ as a possible biomarker of response to chemotherapy in TNBC. 

It is important to address that findings in terms of extent of THRβ expression and cellular location differed across studies. This may be due to difference in patient cohorts (Table 1) and/or technical differences related to the type of antibody used, its concentration and incubation time, as well as variations in interpretation by the reading pathologist. Hence, in future validation studies (possibly multi-centre in nature), it would be critical to ensure that the process of assessment of THRβ is standardized.

Finally, given findings of Heublein et al. THRβ agonists hold promise as potential novel targeted therapies for patients BC [12]. Recently compounds such as Sobetirome (GC-1) and, subsequently, Eprotirome (KB2115) (both THRβ analogs) demonstrated activity as selective thyromimetics. These drugs were halted in phase 1 and phase 2–3 of their development due to harmful alterations observed in dog cartilage following chronic treatment [24]. However, new drugs IS25 and its prodrug TG68 have passed through a comprehensive panel of absorption, distribution, metabolism, and excretion (ADME)-Toxicity and have been identified as very powerful lipid-lowering agents both in vitro and in vivo [24]. Beyond the application of these analogs in dyslipidemia and liver pathologies, the anti-cancer activity of TH analogs with specificity for binding to THRβ1 warrants further evaluation. The anti-cancer activity of THRβ1 agonists is currently being evaluated in cell culture and patient derived xenografts. Pending results of this pre-clinical work, a future phase I clinical trial to explore safety and early efficacy of THRβ1 agonists among patients with advanced solid tumors may be warranted. 

## 5. Challenges/Limitations

Limitations of this review come from inconsistencies between studies, including methodologies used to determine THR expression (IHC versus mRNA levels) and the diverse makeup of various patient cohorts. Further, small sample sizes and the inability to account for all clinically relevant variables (e.g., treatment parameters, follow-up duration) may influence the associations found between THR expression and patient outcome [9,10,12]. Different antibodies used for the immunohistochemical analysis across studies and differences in the IRS score classification may also contribute to variability in reported THR expression levels and predominant localization observed. It should also be noted that six of the seven studies solely focused on patient cohorts with sporadic BCs with limited investigations performed in inherited forms of BC.

Further, thyroid hormone levels were not evaluated in the majority of included studies. Future prospective studies that involve blood collection and assessment of thyroid hormone receptors in tumor tissue would be of interest.

## 6. Conclusions

THRβ expression among patients with early-stage BC is associated with longer disease-free and overall survival in several retrospective cohort studies. The validation of THRβ as an independent prognostic biomarker and its role as a possible predictive biomarker of response to endocrine therapy and/or chemotherapy in prospective studies and ideally in randomized clinical trials is of interest. Given that THRβ is upstream of the AKT/PI3K pathway, its potential as a predictive biomarker of response to AKT inhibitors and/or PI3K inhibitors may also be of value. To address variability in THRβ expression and cellular localization in the literature, future validation studies should require standardization of assessment and central tissue review. Finally, in addition to the potential for THRβ to serve as a prognostic biomarker and predictive biomarker of response to therapy, the potential re-purposing of THRβ agonists as anti-cancer agents warrants investigation.

## Figures and Tables

**Table 1:** Patient cohorts and their characteristics.

Study	Shao et al. [9]	Ditsch et al. [10]	Jerzak et al. [11]	Heublein et al. [12]	Jerzak et al. [4]	Muscat et al. [16]	Gu et al. [17]
Cohort size	n = 271	n = 82	n = 796	n = 124	n = 130	n = 66	n = 1752
Study location	University Hospital, Munich, Germany	Großhadern, Ludwig-Maximilians University, Munich, Germany.	University of Toronto, Toronto, Canada.	Ludwig-Maximilians-University, Munich, Germany.	University of Toronto, Toronto, Canada.	University of Queensland, Queensland, Australia.	Baylor College of Medicine, Texas, USA.
Median age at initial diagnosis (years)	57	68	57	50	65	53 ****	NA
Mean follow-up time (months)	126	144	115	79	NA	NA	87
T stage							
T1	169 (65%)	44 (54%)	65 (8%)	46 (37%)	40 (31%)	NA	614 (30%)
T2	78 (30%)	17 (21%)	289 (36%)	** see notes below	73 (56%)	NA	***
T3	4 (2%)	n/a	390 (49%)	** see notes below	16 (12%)	NA	***
T4	10 (4%)	2 (2%)	48 (6%)	** see notes below	1 (<1%)	NA	***
Tumor grade							
1	13 (9%)	9 (11%)	NA	** see notes below	27 (21%)	NA	208 (10%)
2	95 (63%)	40 (49%)	NA	** see notes below	70 (54%)	NA	***
3	44 (29%)	33 (40%)	NA	77 (62%)	32 (25%)	NA	***
Nodal status							
Positive	112 (44%)	38 (46%)	377 (55%)	66 (53%)	NA	NA	630 (31%)
Negative	144 (56%)	NA	314 (45%)	NA	NA	NA	1034 (51%)
ER status							
Positive	219 (81%)	NA	616 (78%)	55 (44%)	95 (73%)	33 (50%)	1309 (64%)
Negative	53 (19%)		176 (22%)	54 (43%)	NA	33 (50%)	614 (30%)
PR status							
Positive	160 (59%)	NA	479 (60%)	57 (46%)	77 (59%)	NA	378 (19%)
Negative	112 (41%)	NA	313 (40%)	52 (42%)	NA	NA	279 (14%)
HER2 status							
Positive	27 (10%)	NA	219 (31%)	26 (21%)	17 (13%)	NA	NA
Negative	246 (90%)	NA	491 (69%)	51 (41%)	NA	NA	NA
Molecular subtype							
Luminal A (Ki-67 ≤ 14%)	152 (56%)	NA	NA	NA	NA	NA	653 (32%)
Luminal B (Ki-67 > 14%)	60 (22%)	NA	NA	NA	NA	NA	359 (18%)
HER2 positive luminal	20 (7%)	NA	NA	NA	NA	NA	NA
HER2 positive non-luminal	7 (3%)	NA	NA	NA	NA	NA	NA
Triple negative	34 (12%)	NA	101 (13%)	19 (15%)	28 (22%)	NA	NA

NA = Not Available; ER = estrogen receptor; PR = progesterone receptor; HER2 = human epidermal growth factor receptor 2. ** Notes: 17 (44.73%) of BRCA1-associated cases and 61 (70.93%) of sporadic cases had pT2-4 disease. 8 (21%) of BRCA1-associated cases and 38 (44%) of sporadic cases were grade 1 or 2. *** Notes: Data published by Gu et al. were obtained from 2034 samples. 1003 (50%) samples came from patients with T2-4 tumors; 1381 (68%) patient samples came from patients with stage 2–3 BC. **** Notes: Data from Muscat et al. were obtained from 66 samples.

**Table 2:** Studies relating to the expression of THRβ in BCs discussed in this review.

Study	Sample Size	Sporadic vs. Non-Sporadic	Thyroid Receptor Isoform	Method for Biomarker Detection	Receptor Antibody (Working Dilution)	TRβ Predominant Localization	TRβ1 Expression *
Shao et al. [9]	271	Sporadic	THRβ1	THRβ1: Zytomed, 520–4074, Berlin, Germany Scoring: Percent positive cells, intensity, distribution	Anti-THRβ1 (1:200)	Nuclear	Nuclear: 159 (60%), Cytoplasmic: 114 (43%)
Ditsch et al. [10]	82	Sporadic	THRβ1, THRβ2	Immunohistochemistry THRβ1 and THRβ2: Millipore, Schwalbach, Germany Scoring: Percent positive cells, intensity	Rabbit IgG polyclonal: Anti- Anti-THRβ1/2 (1:200), anti-THRβ1 (1:200), Anti-THRβ2 (1:200)	Nuclear	Nuclear: 43 (52%), Cytoplasmic: NA
Jerzak et al. [11]	796	Sporadic	THRβ1	Immunohistochemistry (THRβ1: SC-737 antibody from Santa Cruz Biotechnology, Dallas, TX, USA) Scoring: Percent positive cells, intensity	SC-737 antibody from Santa Cruz Biotechnology (working dilution NA)	Cytoplasmic	Nuclear: NA, cytoplasmic: 318 (40%) high and 478 (60%) Low **
Heublein et al. [12]	124	Sporadic, BRCA1-associated BC	THRβ	Immunohistochemistry (THRβ: Zytomed, Berlin, Germany) Scoring: Percent positive cells, intensity	Anti-THRβ (1:400)	Nuclear	Nuclear and cytoplasmic: NA
Jerzak et al. [4]	130	Sporadic	THRα1, THRα2,	Immunohistochemistry (THRα1: Polyclonal rabbit antibody (ab53729), from Abcam plc; THRα2: Monoclonal mouse antibody (MA1-4676), from Thermo Fisher Scientific Co., Ltd., Waltham, MA, USA)	THRα1 polyclonal rabbit Antibody and THRα2 Monoclonal mouse antibody	Nuclear	NA
Muscat et al. [16]	66	Sporadic: ER+, ER−	THRβ	TaqMan Low Density Gene Signature Arrays (Applied Biosystems, Foster City, CA, USA; catalog item 4379961)	NA	NA	NA
Gu et al. [17]	1752	Sporadic	THRβ	Affymetrix THRβ Probesets (Santa Clara, CA, USA) Cutpoint: 75th Percentile	NA	NA	NA

NA = not available; n/a = not applicable. * Positive expression determined by using immunoreactive score (IRS). ** High expression vs. low expression determined using Allred’s method.

**Table 3:** Clinical associations with THRβ expression.

Study	Sample Size (n)	HR for DFS		HR for OS	
		Univariable	Multivariable	Univariable	Multivariable
Shao et al. [9]	271	Not Significant	NA	Not Reported	Cytoplasmic: 0.545 (*p* = 0.048) Nuclear: 2.860 (*p* = 0.0004)
Ditsch et al. [10]	82	0.41 (*p* = 0.090)	0.83 (*p* = 0.091)	0.55 (*p* = 0.189)	0.97 (*p* = 0.716)
Jerzak et al. [11]	796	Not Reported *	Not Reported	0.46 (*p* < 0.0001)	0.61 (*p* < 0.004)
Heublein et al. [12]	124	NA	NA	KM (*p* = 0.007–0.0189)	NA
Jerzak et al. [4]	130	NA	NA	Cytoplasmic: 0.85 (*p* = 0.19) Nuclear: 1.64 (*p* = 0.63)	Not Calculated
Muscat et al. [16]	66	0.48 (*p* = 0.001)	0.51 (*p* = 0.010)	NA	NA
Gu et al. [17]	1752	NA	NA	KM (*p* < 0.01)	NA

NA = not available; * Significantly associated with recurrence; KM = Kaplan-Meier Analysis; DFS = disease-free survival; OS = overall survival.

## Supplementary Materials

The following supporting information can be downloaded at: https://www.mdpi.com/article/10.3390/curroncol31050176/s1, Table S1: Ovid search.

## Abbreviations

Akt	Protein kinase B
BC	Breast cancer
DFS	Disease-free survival
ER	Estrogen receptor
fT3	Free triiodothyronine
fT4	Free thyroxine
GC-1	Sobetirome
HER2	Human epidermal growth factor receptor 2
HR	Hazard ratio
IC50	Half-maximal inhibitory concentration
IHC	Immunohistochemistry
IRS	Immunoreactive score
JAK-STAT	Janus kinase/signal transducers and activators of transcription
KB2115	Eprotirome
OS	Overall survival
PFS	Progression free survival
PR	Progesterone receptor
RXR	Retinoid X receptor
TH	Thyroid hormone
THR	Thyroid hormone receptor
TRE	Thyroid response element
THRα	Thyroid hormone receptor alpha
THRβ	Thyroid hormone receptor beta
TNBC	Triple negative breast cancer
