# Supplementary material for: The Significance of Thyroid Hormone Receptors in Breast Cancer: A Hypothesis-Generating Narrative Review
Source: Curr Oncol. 2024 Apr 23;31(5):2364–75. doi: 10.3390/curroncol31050176 (PMC11119174; doi:10.3390/curroncol31050176)
Supplement: Supplementary file 1 [file curroncol-31-00176-s001.zip › curroncol-2836293-supplementary.pdf]

Table S1: Ovid search

Ovid Medline search	
1	exp Breast Neoplasms/
2	((breast adj3 cancer*) or (breast adj3 carcinoma*) or (breast adj3 neoplasm*) or breast malignant neoplasm* or breast malignant tumor* or breast neoplasm*).mp.
3	((breast adj3 tumor*) or cancer, breast or cancer of breast or cancer of the breast or human mammary carcinoma* or neoplasm, human mammary or neoplasms, human mammary).mp
4	or/1-3
5	exp Thyroid Hormone Receptors alpha/
6	exp Thyroid Hormone Receptors beta/
7	(THRA* or THR alpha* or TRalpha*).mp.
8	(THRB* or THR beta* or TRbeta*).mp.
9	(ERBA1 Gene Product* or NR1A1 Gene Product* or Proto-Oncogene Protein* c-erbA or Receptor* alpha, Thyroid Hormone or THRA Gene Product* or Thyroid Hormone Receptor alpha-1* or Thyroid Hormone Receptor alpha-2* or c-erb A Protein* or c-erbA Protein* or c-erbA alpha* or c-erbA-1 Protein* or erbA Proto-Oncogene Product*).mp.
10	(ERBA2 Gene Product* or NR1A2 Gene Product* or Receptor* beta Thyroid Hormone or THRB Gene Product* or TR beta* or Thyroid Hormone Receptor beta* or Thyroid Hormone Receptor beta-1* or Thyroid Hormone Receptor beta-2* or c-erbA beta*).mp.
11	or/5-10
12	4 and 11

**Ovid Embase search**	
1	exp breast tumor/
2	((breast adj3 cancer*) or (breast adj3 carcinoma*) or (breast adj3 neoplasm*) or breast malignant neoplasm* or breast malignant tumor* or breast neoplasm*).mp.
3	((breast adj3 tumor*) or cancer, breast or cancer of breast or cancer of the breast or human mammary carcinoma* or neoplasm, human mammary or neoplasms, human mammary).mp.
4	or/1-3
5	exp thyroid hormone receptor alpha/
6	exp thyroid hormone receptor beta/
7	(THRA* or THR alpha* or TRalpha*).mp.
8	(THRB* or THR beta* or TRbeta*).mp.
9	(ERBA1 Gene Product* or NR1A1 Gene Product* or Proto-Oncogene Protein* c-erbA or Receptor* alpha, Thyroid Hormone or THRA Gene Product* or Thyroid Hormone Receptor alpha-1* or Thyroid Hormone Receptor alpha-2* or c-erb A Protein* or c-erbA Protein* or c-erbA alpha* or c-erbA-1 Protein* or erbA Proto-Oncogene Product*).mp.
10	(ERBA2 Gene Product* or NR1A2 Gene Product* or Receptor* beta Thyroid Hormone or THRB Gene Product* or TR beta* or Thyroid Hormone Receptor beta* or Thyroid Hormone Receptor beta-1* or Thyroid Hormone Receptor beta-2* or c-erbA beta*).mp.
11	5 or 6 or 7 or 8 or 9 or 10
12	4 and 11
